# Tear Fluid Catecholamines As Biomarkers of the Parkinson’s Disease: A Clinical and Experimental Study

**DOI:** 10.32607/20758251-2019-11-4-99-103

**Published:** 2019

**Authors:** A. R. Kim, M. R. Nodel, T. A. Pavlenko, N. B. Chesnokova, N. N. Yakhno, M. V. Ugrumov

**Affiliations:** Koltzov Institute of Developmental Biology of Russian Academy of Sciences, Moscow, 119334 Russia; Sechenov First Moscow State Medical University, Moscow, 119991 Russia; Pirogov Russian National Research Medical University, Russian Clinical and Research Center of Gerontology, Moscow, 129226 Russia; Helmholtz Moscow Research Institute of Eye Diseases of Ministry of Health of the Russian Federation, Moscow, 105062 Russia

**Keywords:** Parkinson’s disease, tear fluid, patients, experimental models, biomarkers

## Abstract

An important approach to an early diagnosis of Parkinson’s disease (PD) is screening for peripheral biomarkers in patients at the early clinical stage. In this study, we evaluated catecholamine concentration changes in the tear fluid of untreated PD patients as biomarkers. Norepinephrine and dopamine concentrations in the tear fluid of patients were found to increase compared to those in age controls, which was especially pronounced on the side where motor symptoms appeared. On the contrary, the epinephrine concentration in the tear fluid of patients was reduced bilaterally. Since there was no reason to consider the markers found in the clinical stage of PD as markers of the preclinical stage, we additionally studied the tear fluid composition in mouse neurotoxic models of PD preclinical and clinical stages. The norepinephrine concentration in the tear fluid of mice from the clinical stage model was found to be higher than that in controls; in the preclinical stage model, the norepinephrine concentration had a tendency to increase. Therefore, both PD patients and mice from PD preclinical and clinical stage models manifest unidirectional changes in their tear fluid compositions, which may be considered as promising biomarkers for the development of early diagnosis.

## INTRODUCTION

Parkinson’s disease (PD) is a common neurodegenerative disorder that is characterized, in particular, by the death of the dopaminergic neurons of the brain nigrostriatal system. Clinically, PD manifests itself many years after the disease onset, after most of the nigral dopaminergic neurons have died, which explains the limitations of the current PD pharmacotherapy [1]. Therefore, the challenge is to develop an early (preclinical) diagnosis of PD, which would enable detection of the disease before the appearance of the first motor symptoms and early start of preventive neuroprotective therapy [1].

In recent years, growing attention has been focused on the investigation of visual system changes in PD and the underlying pathological processes in the eye and accessory visual structures, which may be potential sources of peripheral PD biomarkers [2]. An important role is played by the impaired metabolism of the catecholamines that ensure the transmission of visual information in the retina and regulate the accommodation rate and intraocular pressure [2]. In addition, PD is accompanied by changes in the eyelid tissue containing numerous glands whose secretory products form the tear fluid. The conjunctiva lining the inner surface of the eyelids and conjunctival glands display sympathetic innervation [3]; dysfunction of the conjunctiva occurs in PD as part of a multisystem degeneration affecting both the central and peripheral parts of the autonomic nervous system [4].

Tear fluid sampling is a simple non-invasive procedure, in contrast to the blood or cerebrospinal fluid sampling traditionally used to screen for peripheral biomarkers [5]. However, only a few studies have been focused on a search for PD biomarkers in the tear fluid, with only the protein composition being analyzed. For example, the tear fluid of PD patients has elevated levels of the tumor necrosis factor [6] and oligomeric α-synuclein [7], as well as a changed proteomic profile in general [8]. These studies indicate that there is a prospect of searching for PD biomarkers in the tear fluid. However, it makes sense to analyze not only proteins, but also the low-molecular substances involved in the PD pathogenesis, in particular the catecholamines that have been actively studied as potential blood and cerebrospinal fluid biomarkers of PD [9].

It should be emphasized that there is no data indicating that the biomarkers found in patients with a diagnosed clinical stage of PD could be related to the preclinical stage of this disease [1]. Therefore, we performed a comparative analysis of catecholamine content changes in the tear fluid of untreated PD patients at the early clinical stage and animal models of the preclinical and clinical stages of PD.

## MATERIALS AND METHODS

**PD patients and a control group**

We analyzed tear fluid samples from 26 PD patients at Hoehn-Yahr stage 1–2 before the start of anti-parkinsonian therapy and from subjects of similar age without motor impairment. All patients gave written consent to participate in the study.

The PD diagnosis was made in accordance with the 2015 Movement Disorder Society (MDS-2015) clinical diagnostic criteria. The control group included individuals of the same age without neurological diseases. Patients with ophthalmic diseases were not included in the study. The key clinical characteristics of the cohorts are presented in *Table 1*.

**Table 1:** Clinical characteristics of patient cohorts

Cohort	N	Gender, m/f	Age, years	PD stage assessment			Disease duration, years
				Hoehn-Yahr scale	UPDRS II (daily activity)	UPDRS III (motor activity)	
PD patients	26	16/10	60.3 ± 2.0	1.8 ± 0.1	8.7 ± 1.0	23.6 ± 2.3	2.6 ± 0.3
Control	19	4/15	57.4 ± 2.9	–	–	–	–

The tear fluid was collected in the morning using sterile filter paper (5 mm wide) that was placed behind the lower eyelid, as in the Schirmer test. The tear fluid was collected by natural sorption on a test strip, without lacrimation stimulation, for 5 min. The length of the moistened strip was measured to calculate the sample volume, after which the strips were placed in test tubes with 0.1 N HClO_4_, frozen in liquid nitrogen, and stored at –70°C.

**Animals**

We used 30 male C57BL/6 mice aged 2–2.5 months and weighing 22–26 g (Pushchino nursery). The animals were kept under standard conditions with free access to food and water. PD at the preclinical stage was modelled by two subcutaneous injections of 1-methyl-4-phenyl-1,2,3,6-tetrahydropyridine (MPTP) (Sigma, USA) at a single dose of 8 mg/kg. PD at the clinical stage was modelled by four subcutaneous MPTP injections at a dose of 10 mg/kg. The interval between the injections in both models was 2 h [10]. The control group received 0.9% NaCl according to a similar schedule.

Two weeks after administration of MPTP or 0.9% NaCl, the condition of the mice was assessed by the distance traveled in an open field test using a PhenoMaster animal behavior analysis system (TSE Systems, Germany). Then, the tear fluid was collected from the animals under isoflurane anesthesia, using 2.5-mm wide filter paper strips similar to Schirmer’s strips.

After collecting the tear fluid, the anesthetized mice were decapitated and the dorsal striatum was dissected from the brain according to the previously described technique [10]. Striatum samples were weighed, frozen in liquid nitrogen, and stored at –70°C.

**High Performance Liquid Chromatography (HPLC)**

The concentration of catecholamines (norepinephrine, epinephrine, and dopamine) was measured using high-performance liquid chromatography with electrochemical detection (HPLC-ED). Samples were homogenized using a Labsonic M ultrasonic homogenizer (Sartorius, France) in 200 μL of 0.1 N HClO_4_ (Sigma, USA) containing an internal standard of 25 pM/mL 3,4-dihydroxybenzylamine (DHBA, Sigma) and centrifuged at 2 000 *g *for 20 min.

HPLC was performed on a ReproSil-Pur ODS-3 reversed-phase column, 4 × 100 mm, 3 μm pore size (Dr. Majsch GmbH, Germany), at a temperature of +30°C and a mobile phase rate of 1.2 mL/min using a LC-20ADsp liquid chromatograph (Shimadzu, Japan), as described previously [11].

**Statistics**

The HPLC data are presented as percentage means (normalized to control) ± standard error of the mean. Since PD develops asymmetrically, the data collected from the patients were allocated into ipsilateral side data (tear fluid samples collected from the eye on the side where motor symptoms appeared and had greater severity) and contralateral side data (samples collected on the side where motor symptoms were absent or had mild severity). In the control group and experimental animals, the data obtained from the analysis of the tear fluid from the right and left eyes were averaged.

Normality of the data was examined using the Shapiro–Wilk test. Statistical analysis of the data was performed by the parametric Student *t*-test or the non-parametric Mann–Whitney test, using the GraphPad Prism 6.0 software (GraphPad Software, USA). The significance criterion was *p *≤ 0.05.

## RESULTS AND DISCUSSION

**Fig. 1:**
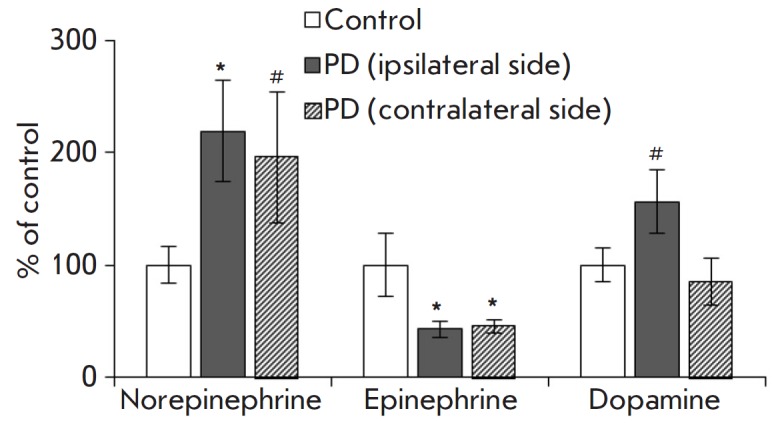
Catecholamine concentration in the tear fluid of PD patients sampled from the eye on the side of motor symptom appearance (ipsilateral) or on the opposite side (contralateral). * *p *≤ 0.05, # *p *≤ 0.05 relative to the control

In this work, the catecholamine concentration in the tear fluid of untreated PD patients was measured for the first time. The norepinephrine and dopamine levels in the tear fluid of patients were shown to be significantly higher than in healthy subjects (controls) of the same age (*Fig. 1*). An important asymmetry was found: the increase in the norepinephrine and dopamine concentrations was more pronounced on the ipsilateral side (on the side of more severe motor symptoms), compared to the contralateral side. Because there were no statistically significant differences in the volume of the collected samples (data not shown), the revealed asymmetry in the dopamine and norepinephrine concentrations in PD patients cannot be explained by asymmetric hypokinesia of the eye muscles with an impaired tear outflow. However, the asymmetry of marker content changes in the tear fluid is in good agreement with the known facts regarding the asymmetric nature of PD development. For example, in the early clinical stage of PD, the threshold degradation of the nigrostriatal dopaminergic system and motor disorders appear only on one side [12]. It should be emphasized that tear composition asymmetry is the first observation of asymmetric PD development in the peripheral organs [12].

Unlike norepinephrine and dopamine, the epinephrine level in the tear fluid decreased on both the ipsilateral and contralateral sides (*Fig. 1*). This response is similar to the previously established decrease in plasma epinephrine in PD patients [9]. It should be noted that the source of catecholamines in the tear fluid is not exactly known. It is known that epinephrine enters the bloodstream from the adrenal glands, while norepinephrine and dopamine mainly originate from sympathetic noradrenergic nerve terminals [13]. Probably, the catecholamines found in the tear fluid are of similar origin.

**Fig. 2:**
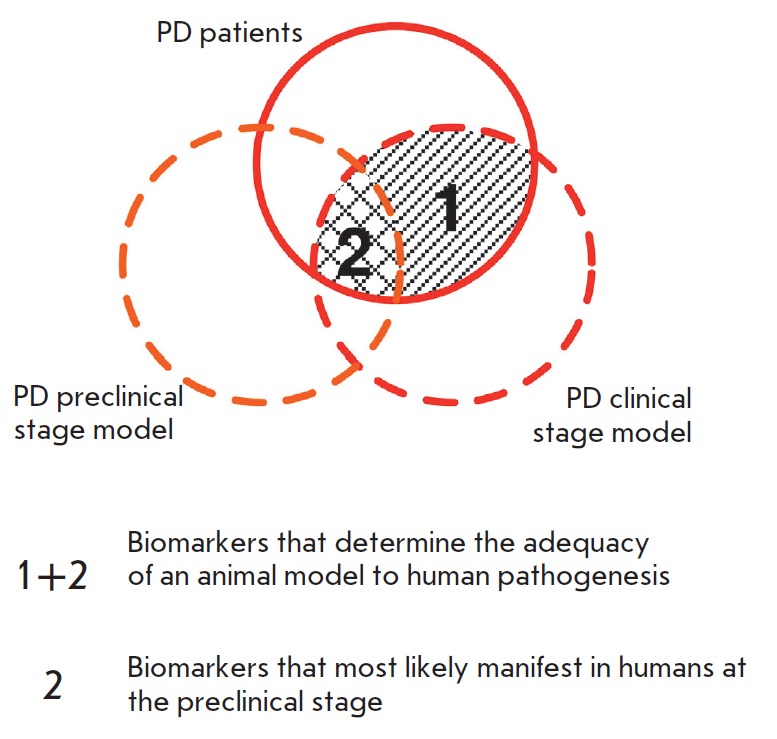
Schematic of the biomarker test methodology

The catecholamine concentration changes in the tear fluid of PD patients could potentially be used to develop an early diagnosis. However, there is always a risk that the biomarkers detected in patients at the clinical stage are absent at the preclinical stage. In this regard, experimental modeling of PD is of particular value because it may be used to reproduce both stages of the disease [1, 10]. For example, according to our general methodology, matching of the biomarkers found both in patients and in animal models of PD clinical stage indicates a correct reproduction of these aspects of the disease pathogenesis (*Fig. 2*). Some of them may be considered as biomarkers of a PD preclinical stage if they are also detected in a preclinical stage model [9].

An important feature of PD is the precisely defined neurodegeneration threshold, surpassing of which causes motor symptoms: death of 50–60% of dopaminergic neuronal bodies in substantia nigra, loss of 70–80% of dopaminergic neuron axons in the striatum, and, accordingly, a decrease in the striatal dopamine level by 70–80% compared to the control [1].

**Fig. 3:**
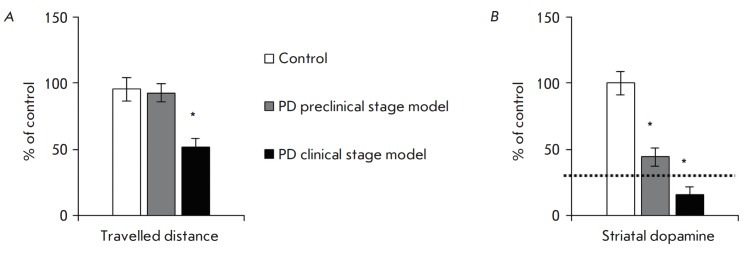
Total distance in the open-field test (*A*) and the dopamine concentration in the striatum (*B*) of control mice and mice from PD preclinical and clinical stage models. * *p *≤ 0.05 in comparison to control mice; dotted line – threshold of motor symptom appearance

According to our findings, 2 weeks after the double administration of MPTP at a dose of 8 mg/kg, mice showed no changes in the open field test, and the striatal dopamine level decreased by 65.6% (*Fig. 3*). In turn, in animals that received four injections of 10 mg/kg MPTP, the traveled distance parameter decreased by almost half and the striatal dopamine level dropped by 83.3% (*Fig. 3*). Therefore, the reproduced models of PD preclinical and early clinical stages fully correspond to the key parameters described above.

**Fig. 4:**
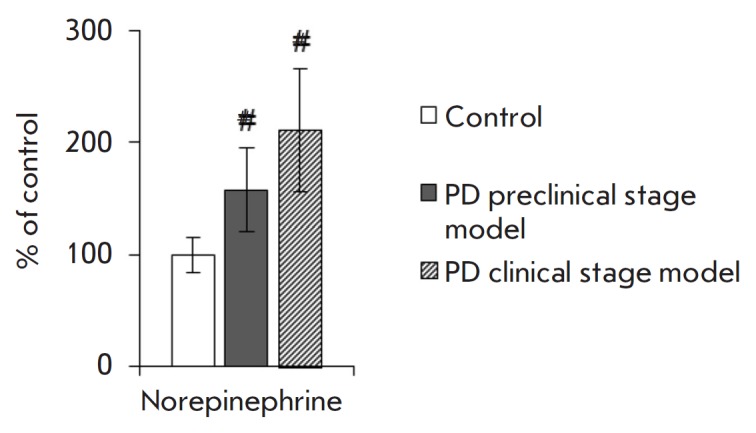
Norepinephrine concentration in the tear fluid of control mice and mice from PD preclinical and clinical stage models. # *p *≤ 0.05 in comparison to control mice

The norepinephrine concentration in the tear fluid of mice had a tendency (p < 0.15) to increase by 57.6% in the PD preclinical stage model and by 111% in the PD clinical stage model compared with that in the control (*Fig. 4*). The epinephrine and dopamine levels were below the detection limit, which is most likely due to the small volume of the collected samples (1 to 2 μL). There are methods to stimulate lacrimation in animals using cholinomimetics [14], but the composition of stimulated tear fluid is known to vary significantly [15].

Comparison of the changes in the levels of norepinephrine, epinephrine, and dopamine in the tear fluid of PD patients and in mouse PD models revealed an increase in the norepinephrine concentration in all three cases, compared to that in the controls (*Table 2*).

**Table 2:** Comparative analysis of biomarkers

Biomarker	PD patients	Mouse PD models	
		Clinical stage	Preclinical stage
Norepinephrine	↑	↑#	↑#
Epinephrine	↓	ND	ND
Dopamine	↑#	ND	ND

Note. ↑, ↓ – an increase and decrease, respectively, in the biomarker concentration in tear fluid compared to the control, ND – not determined, # – tendency (p < 0.15).

Therefore, an increase in the norepinephrine concentration in the tear fluid was found in both PD patients and mouse models of PD preclinical and clinical stages. This may be considered as a promising biomarker for an early diagnosis of PD.

## Abbreviations

DHBA: 3,4-dihydroxybenzylamine
HPLC-ED: high-performance liquid chromatography with electrochemical detection
MPTP: 1-methyl-4-phenyl-1,2,3,6-tetrahydropyridine
PD: Parkinson’s disease
